# In vitro fertilization with frozen embryo transfer increased histamine-mediated contractile sensitivity via PKCβ in human umbilical vein

**DOI:** 10.1186/s12958-023-01103-8

**Published:** 2023-06-13

**Authors:** Jiaqi Tang, Linglu Qi, Yun He, Na Li, Ze Zhang, Xiuwen Zhou, Hongyu Su, Qiutong Zheng, Yumeng Zhang, Jianying Tao, Zhice Xu

**Affiliations:** 1 Institute for Fetology, First Hospital of Soochow University, Suzhou, Jiangsu China; 2 Maternal and Child Health Care Hospital of Wuxi, Wuxi, Jiangsu China; 3 Women’s Hospital School of Medicine Zhejiang University, Zhejiang, China; 4 Department of Obstetrics and Gynecology, Taixing People’s Hospital, Taixing, Jiangsu China; 5 Suzhou Municipal Hospital, the Affiliated Suzhou Hospital of Nanjing Medical University, Jiangsu, China

**Keywords:** Frozen embryo transfer, Histamine, In vitro fertilization, Umbilical vein, Vascular tension

## Abstract

**Objective:**

In vitro fertilization-embryo transfer (IVF-ET) technologies (especially frozen ET) have been widely used, which might affect maternal and fetal health. Information regarding influence of IVF-ET on the vasoconstriction of human umbilical vein (HUV) is limited. This study determined effects of frozen ET on histamine-mediated vascular responses in HUV and related mechanisms.

**Methods and results:**

HUVs were collected from frozen ET conceived pregnancy and spontaneously conceived pregnancy (control). Histamine concentration in umbilical plasma was higher in frozen ET group than the control. Histamine-mediated contractile response curve was left-shifted in the frozen ET group when comparing with the control. In isolated HUV rings, H1R showed a critical role in regulating vascular constriction, while H2R played little roles in regulating vessel tone. Iberiotoxin and 4-aminopyridine didn’t significantly change histamine-mediated constriction in HUVs. Histamine-induced vasoconstrictions were significantly decreased by nifedipine, KN93, or GF109203X, while the inhibitory effects were significantly greater in the frozen ET group in comparison to the control. The constrictions by Bay K8644, phenylephrine, or PDBu were stronger in frozen ET, respectively. There was a decrease in the protein expressions of H1R and H2R, an increase in protein expressions of BK_Ca_α and PKCβ.

**Conclusions:**

Histamine-induced constriction in HUV was mainly via H1R. The increased sensitivity to histamine in HUV following frozen ET cycles were linked to the enhanced PKCβ protein expression and function. The new data and findings in this study provide important insight into influences of frozen ET on fetal vessel development and potential influence in long-term.

**Supplementary Information:**

The online version contains supplementary material available at 10.1186/s12958-023-01103-8.

## Introduction

More than 8 million babies have been born following in vitro fertilization-embryo transfer (IVF-ET) in the world since 1978 [1]^,^ [2]. Assisted reproductive technologies involve embryo transfer cycles [3, 4], which consist of the fresh or frozen ET cycles [4]. Over the last decade, the use of frozen ET cycles has dramatically risen [5]. Although the great majority of children born through IVF-ET are healthy, some reports have indicated the potential risks of adverse perinatal conditions [6]. Based on the developmental origins of health and diseases hypothesis (adverse perinatal environment can affect the development and increase risks of cardiovascular and metabolic diseases in adulthood) [6, 7], there are concerns about perinatal risks of IVF-ET on fetuses as well as long term effects on children [8].

Notably, umbilical vessels play a vital role in feto-placental circulation [9]. Due to lack of autonomic innervation, vessel tone of human umbilical vessels is mainly regulated by vasoactive substances released locally or carried by the blood stream, such as histamine [10, 11]. Recent studies reported that IVF-ET could alter the constriction caused by angiotensin II and acetylcholine in the HUVs via abnormal DNA methylation [12, 13]. However, there have been very limited data on the effects of frozen ET process on vascular functions in human. Considering that the umbilical cord is only organ or tissue that can be obtained and used ethically from healthy human fetuses for scientific research and umbilical veins (HUV) are part of fetal vessels, this study determined possible effects of frozen ET on vascular functions in HUV.

It is well known that histamine can cause reliable and strong contractions in human umbilical vessels from non-IVF pregnancies [14]. Histamine participated in cell proliferation and differentiation during pregnancy [15]. The biogenic amine histamine discovered in 1910 can mediate vascular constriction mainly via H1R, as well as dilation via H1R and/or H2R in most vessels [16, 17]. In human umbilical arteries from non-IVF pregnancies, histamine induced contractions through H1R, while H2R activation led to relaxation [18]. However, it is still unknown whether and how H1R and H2R participate in regulating the vessel tone of human umbilical veins. This study tested vessel tone regulated by histamine in HUV and related receptors, as well as compared vascular responses to histamine in HUV between the control and frozen ET groups.

Ion channels, especially potassium channels and calcium channels play vital roles in regulating vessel tone [10]. K_V_ channels and BK_Ca_ channels are predominant in umbilical vessels [11, 19]. Our laboratory previously reported BK_Ca_ channels participated in acetylcholine-mediated constriction in HUV [20]. The alterations in the expression or functional activities of BK_Ca_ and K_V_ channels were associated with pregnancy-induced pathologies, such as preeclampsia [10, 11, 21]. This study tested possible roles of both potassium channels in histamine-mediated constriction in HUV.

Besides, L-type voltage-dependent calcium channels (CACNA1C) are determinant for umbilical vessel tone [10, 22]. Nifedipine could significantly inhibit vasoconstriction in HUV [23, 24]. Calcium/calmodulin-dependent protein kinase 2 (CAMK2) is a critical regulator in vessel tone [25]. Histamine could evoke the increase of intracellular calcium in smooth muscle cells [26]. Roles of CACNA1C and CAMK2 are unknown in histamine-mediated vascular effects in HUV. Therefore, the functions of ion channels and CAMK2 in histamine-mediated contractile responses were tested in HUV from both groups.

Protein kinase C (PKC) participates the regulation of vessel tone [27, 28], consisting three kinds of isoforms, such as conventional calcium dependent isoforms α and β. Activated PKC can phosphorylate ion channels so as to regulate vessel tone [29]. Pan-inhibitor for PKC, GF109203X could partially decrease acetylcholine-mediated constriction in HUV from non-IVF pregnancies, demonstrating its vital role in regulating umbilical vessel tone [12]. The present study determined vascular tension using the agonist and inhibitor for PKC following frozen ET treatments.

Different from previous reports (comparison between IVF-ET and the control), this study focused on effects of IVF-frozen embryo transfer on vessel tone of HUV. Possible roles of ion channels, CAMK2, and PKC were determined in histamine-induced constriction in HUV, which would provide new insight into influence of frozen ET on fetal vessel development.

## Materials and methods

### Ethics approval and sample collection

All procedures used in this study were approved by the Institute’s Ethics Committee of First Hospital of Soochow University (NO. 278–2020). All participants in this study were informed of consent. Human umbilical cords and umbilical blood were collected following caesarean section or spontaneous vaginal delivery. Inclusion criteria: woman with singleton and no obvious complicating diseases (such as gestational hypertension and gestational diabetes). The characteristics of the participants were listed in Table 1. The umbilical cords were obtained from normal pregnancy (Ctrl, N = 22) and the pregnancy that received frozen embryo transfer (frozen ET, N = 20) and kept at 4℃ in Krebs solution.

**Table 1:** Characteristics of pregnancy

Groups	Age (years)	Systolic blood pressure (mmHg)	Diastolic blood pressure (mmHg)	Gestational weeks		Body weight of newborn (g)	N
Ctrl	32.14 ± 4.18	118.60 ± 12.20	71.71 ± 8.90	39.03 ± 0.54	3452 ± 298		22
frozen ET	31.33 ± 4.80	122.50 ± 12.84	76.46 ± 8.52	39.07 ± 0.89	3386 ± 373		20

### Measurement of the vascular tension

The human umbilical veins (HUV, diameter: about 1,200 μm, lumen diameter: about 760 μm) were carefully isolated and cut into rings, approximately 4–5 mm in length. HUV rings were suspended in a 5 mL organ bath with Krebs solution. The vessel tone was measured with isometric force transducer and recorded using Med-Lab6. Tissue baths were maintained at 37℃, and gassed continuously with a mixture of 95% O_2_ and 5% CO_2_. Vascular rings were given 2 g of initial tension and allowed to equilibrate for 1-2 h. Then the rings were stimulated with 120 mmol/L potassium chloride (KCl) to achieve maximal tension. Vascular contraction was normalized to the maximal tension elicited by KCl. Accumulative concentration of histamine (10^− 9^-10^− 3^ mol/L) was added into the organ bath to obtain the dose-response curves. There was at least a 3 min interval between successive doses of histamine to reach plateau phase of the response. HUV rings were incubated with iberiotoxin (IbTX, BK_Ca_ channel specific blocker, 10^− 7^ mol/L), 4-aminopyridine (4AP, K_V_ channel blocker, 10^− 3^ mol/L), nifedipine (nife, L-type calcium channel blocker, 10^− 5^ mol/L), KN93 (CAMK2 inhibitor, 10^− 5^ mol/L), diphenhydramine (PHR, H1R inhibitor, 10^− 5^ mol/L), or famotidine (FA, H2R inhibitor, 10^− 5^ mol/L), respectively, before application of histamine.

### Real-time PCR

Total RNA was extracted from HUV tissues using Trizol reagents (Takara). The purity and integrity of the RNA were determined with Nanodrop and agarose gel electrophoresis. RNA was reversely transcribed into cDNA using revert aid first-strand cDNA synthesis kit. All gene primer sequences were shown in Table 2. Real-time PCR was performed with the SYBR green supermix Taq kit and analyzed on an iCycler, MyiQ two color real-time PCR detection systems (Bio-Rad). The 2^−ΔΔCt^ method was used to comparatively quantify the mRNA levels of target genes and β-actin was used as an endogenous reference gene.

**Table 2:** Primer sequences

Gene name	Forward Primer	Reverse Primer
*ACTB*	GGACTTCGAGCAAGAGATGG	AGCACTGTGTTGGCGTACAG
*CACNA1C*	CCAAGGAGGAGAAGATTGAGC	GAAGCTCAGAGAGTGGTCGTG
*CAMK2D*	ATCCCAGAAGGGTGGATACCC	GGCAGACTTTGGCTTAGCCATAG
*CAMK2G*	GGCTTCAGGAGTTACCGTGTC	ATCCCTATGGAAAACCTGTGG
*HRH1*	ACAAGATGTGTGAGGGCAACAAG	GTACGGCATACAGCACCAGC
*HRH2*	CATCACCGTGGTCCTTGC	AGCTGGTAGATGGCAGAGAAGG
*KCNMA1*	GGTCTCCTGGGAGTCAACATT	GCTGAGAGATGCTCACCTCAG
*KCNMB1*	AATAGGACGCTGGTTTCGTTC	TGTACCACACGGAGGACACTC
*PRKCA*	GACGCAGCTGCACTCCTG	CGATGGAAATCTCTGCCG
*PRKCB*	GAATCGGACAAAGACAGAAGACTG	GGAGACAGTGTTGGTCGTCTT

### Western blot

HUV were homogenized in liquid nitrogen and thawed in RIPA buffer containing protease inhibitors to obtain the whole cell lysates. The protein abundances of H1R, H2R, BK_Ca_α, BKCaβ1, CACNA1C, CAMK2D, PKCα and PKCβ in the HUV were normalized to GAPDH. The primary antibodies for H1R (Cohesion Biosciences, CPA7350), H2R (Cohesion Biosciences, CPA1551), BK_Ca_α (Santacruz, sc-374,142), BK_Ca_β1 (Immunoway, YN0522), CACNA1C (Proteintech, 21774-1-AP), CAMK2D (Proteintech, 20667-1-AP), PKCα (Cohesion Biosciences, CPA5525), and PKCβ (Proteintech, 12919-1-AP) were incubated overnight at 4℃. Following washing with Tris-buffered saline containing 0.2% Tween 20, the membranes were incubated with secondary antibodies (Multisciences, GAM0072 or GAR0072) for 1 h. The immunoreactive bands were visualized using Tanon UVP imaging system. Imaging signals were digitized and analyzed, and the ratio of the band intensity to GAPDH was subsequently obtained to quantify the relative protein expression.

### Histamine ELISA in umbilical plasma

A commercial enzyme-linked immunosorbent assay kit was used to quantify concentration of histamine in umbilical plasma. The procedure was performed according to the instructions. The umbilical plasma was added to the wells coated with purified human histamine antibody and labeled with horseradish peroxidase. Following washing completely, tetramethylbenzidine substrate solution was added. The reaction was terminated by addition of the sulphuric acid solution and color changes were measured with microplate reader at a wave-length of 450 nm. The concentration of histamine was then determined by comparing the O.D. value of samples to the standard curve.

### Statistical analysis

Data were expressed as the mean ± S.D. Statistical significance (p < 0.05) were determined by unpaired t-test or two-way ANOVA with repeated measures followed by a Bonferroni *post hoc* test. GraphPad Prism (version 8.0) was used to analyze the dose-dependent responses.

## Results

### Histamine mediated vasoconstrictions in human umbilical veins

**Fig. 1:**
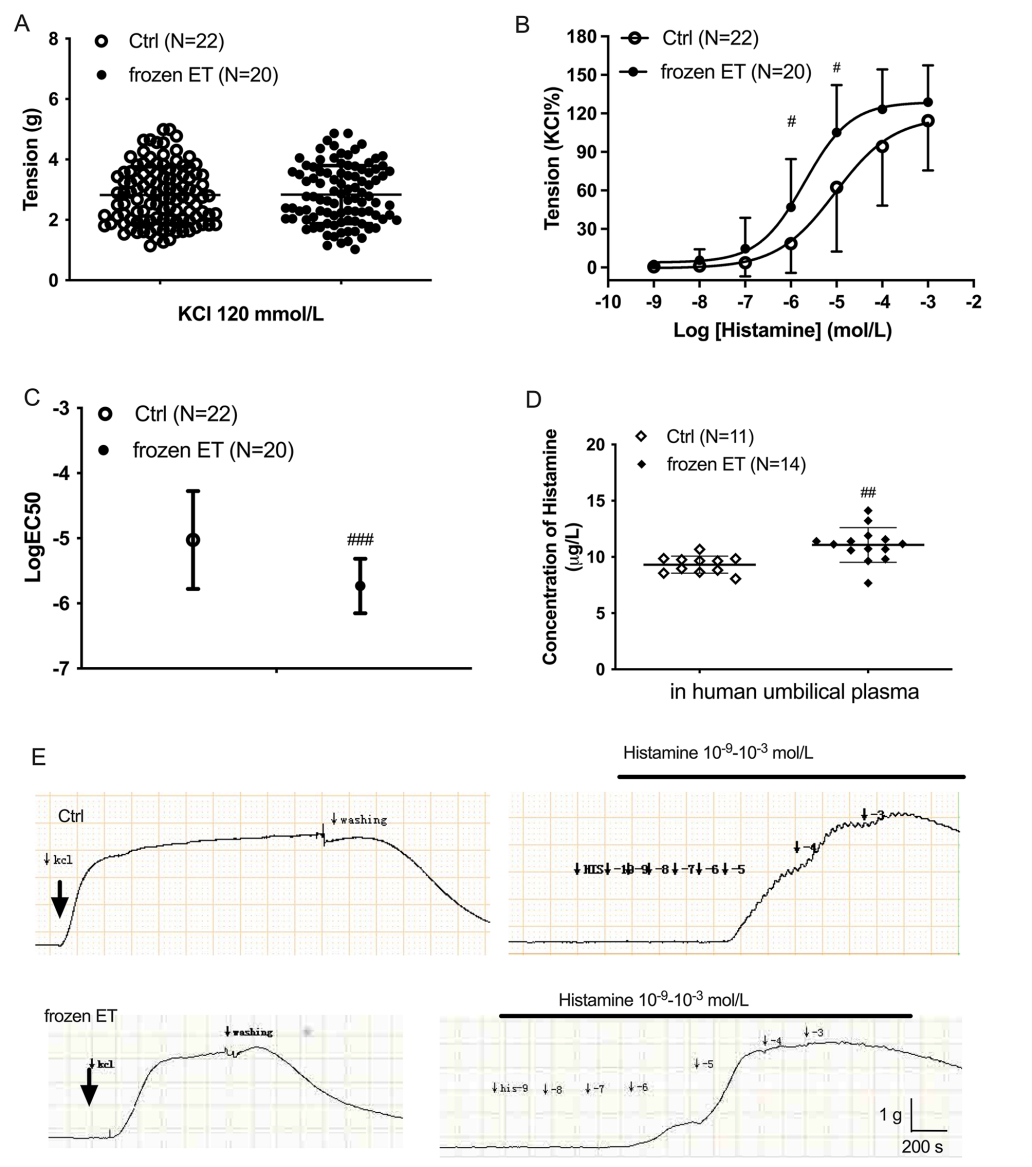
Histamine-mediated contractile responses in HUV from normal pregnancy and the pregnancy who received frozen embryo transfer (**A**) Potassium chloride (KCl)-mediated constrictions in HUV. (**B**) Contractile responses to accumulated concentrations of histamine in HUV. (**C**) LogEC50 of the contractile responses caused by histamine in HUV. (**D**) The concentration of histamine in human umbilical plasma. (**E**) The real-time records of histamine-induced constriction in HUV. Ctrl, normal pregnancy. Frozen-ET, the pregnancy who received the frozen embryo transfer. Inter-group comparison, ^#^, p < 0.05; ^##^, p < 0.01; ^###^, p < 0.001

The maximum constrictions caused by KCl in HUV were comparable among control group and frozen ET group (Fig. 1A). Histamine caused dose-dependent constrictions in human umbilical veins from control pregnancy and the pregnancy who received frozen ET (Fig. 1B). Histamine at the doses of 10^− 6^ and 10^− 5^ mol/L caused significantly stronger contractions in frozen ET group than that in the control (Fig. 1B). The value of LogEC50 in frozen ET group was lower than the control group. These results indicated that the umbilical veins from the frozen ET group were more sensitive to histamine than those from the control (p < 0.001). Figure 1E presents the real-time record of histamine-mediated constriction in human umbilical veins from both groups.

### Histamine concentration in umbilical plasma

Figure 1D shows that histamine concentration in umbilical plasma was 11.07 ± 1.53 µg/L in frozen ET group, which was higher than that in the control (9.31 ± 0.76 µg/L). With considering the molecular weight of histamine, the concentration of histamine in human umbilical plasma is near 10^− 7^ mol/L. The tension (%KCl) caused by histamine at 10^− 7^ mol/L in normal umbilical vein is 3.62 ± 2.26 (mean ± SEM), relatively lower than that (14.60 ± 5.38) in frozen ET umbilical vein. The higher histamine concentration in umbilical plasma from the frozen ET group might potentiate the contractile responses.

### Role of histamine receptors in HUV

**Fig. 2:**
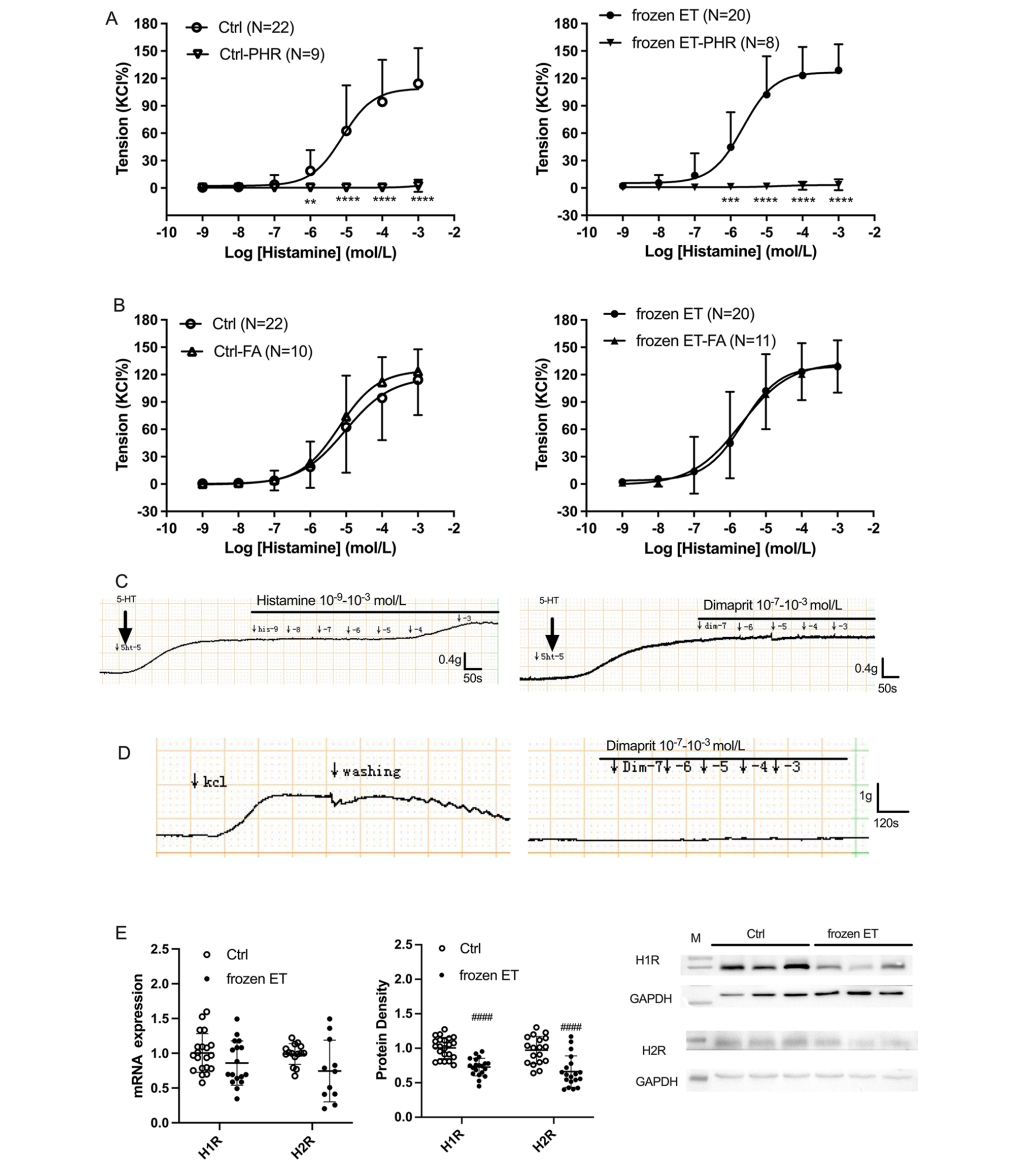
Functions of H1R and H2R in histamine-induced vasoconstrictions of HUV (**A**) Histamine-induced constrictions in HUV in the presence or absence of diphenhydramine (PHR, an H1R inhibitor, 10^− 5^ mol/L). (**B**) Histamine-induced constrictions in HUV with or without famotidine (FA, an H2R inhibitor, 10^− 5^ mol/L). (**C**) Real-time records of doses of histamine or dimaprit-mediated vascular responses following 5-HT. (**D**) Real-time record of dimaprit-mediated vascular response in HUV. (**E**) The mRNA and protein expressions of H1R and H2R. Intra-group comparison, **, p < 0.01; ***, p < 0.001; ****, p < 0.0001. Inter-group comparison, ^####^, p < 0.0001

Diphenhydramine (PHR, an H1R inhibitor) significantly inhibited the contractile response to histamine in HUV in two groups (Fig. 2A). However, famotidine (FA, an H2R inhibitor) had no significant influence on histamine-induced response curve in both groups (Fig. 2B). These indicated histamine-caused constriction in HUV was predominantly via H1R, instead of H2R. In addition, Fig. 2C shows that histamine and the H2R agonist (dimaprit dihydrochloride, Dim) did not obviously dilate vessel tone following serotonin-induced contractile plateau, suggesting that H2R played little role in regulating HUV dilation. Additionally, dimaprit dihydrochloride did not caused a significant contraction in HUV (Fig. 2D), demonstrating that H2R played little role in regulating vessel tone of HUV. Figure 2E presents there was a decrease in H1R and H2R protein expressions in frozen ET when comparing with the control, while mRNA expressions were comparable between two groups.

### Role of potassium channels in histamine-induced contraction in human umbilical veins

**Fig. 3:**
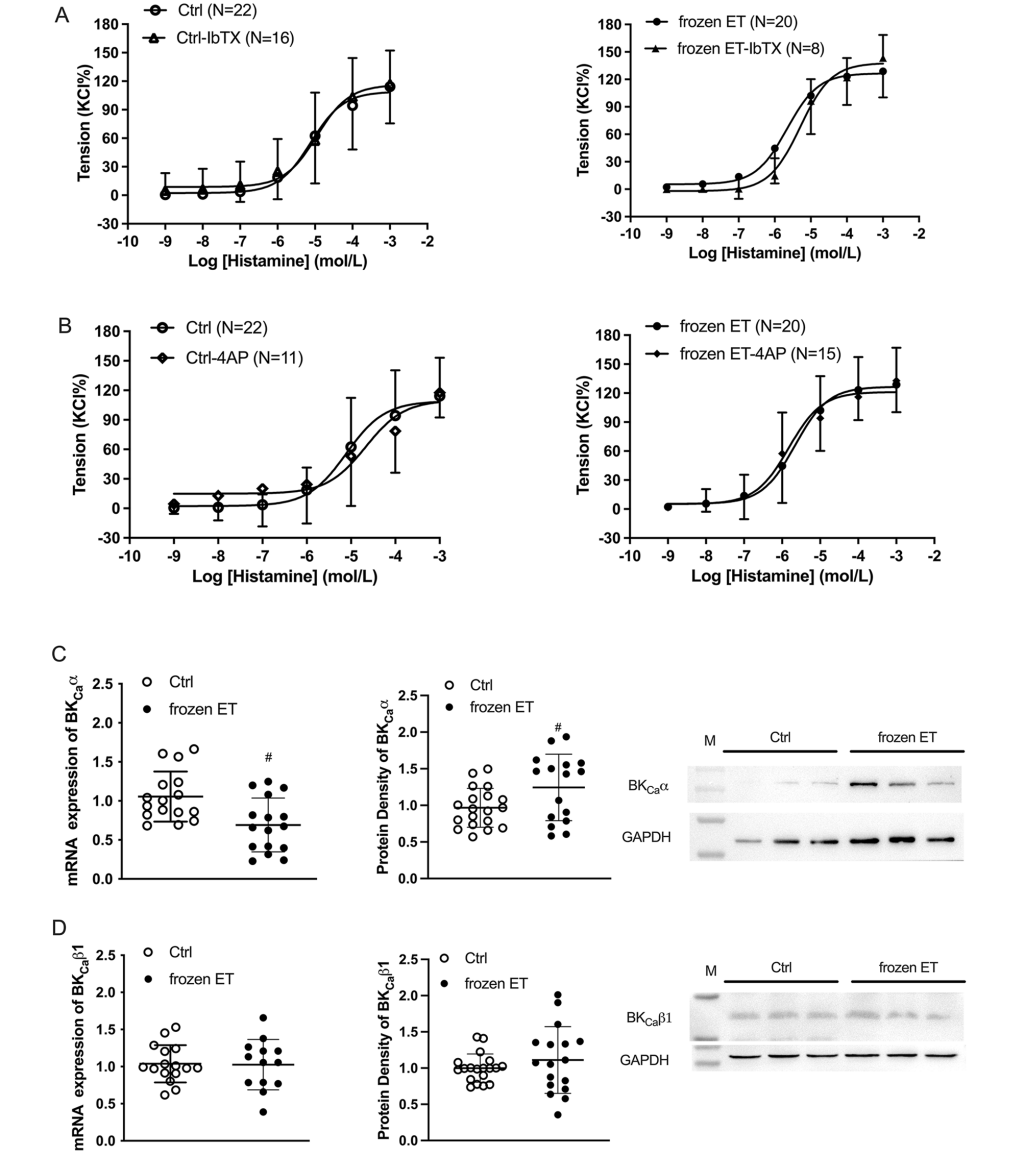
Functions of BK_Ca_ and K_V_ channels in histamine-induced vasoconstrictions in HUV (**A**) The concentration response curves to histamine in the presence or absence of iberiotoxin (IbTX, a specific BK_Ca_ channels blocker, 10^− 7^ mol/L). (**B**) The concentration response curves to histamine in the presence or absence of 4-aminopyridine (4AP, a K_V_ channel blocker, 10^− 3^ mol/L). (**C-D**) The mRNA and protein expressions of BK_Ca_ (α and β1). Inter-group comparison, ^#^, p < 0.05

Figure 3 A and 3B show that histamine-induced vasoconstrictions were not significantly changed by iberiotoxin (IbTX, a selective BK_Ca_ channel inhibitor) or 4-Aminopyridine (4AP, a K_V_ channel inhibitor) in both groups, illustrating that BK_Ca_ and K_V_ channels played little role in the regulations of histamine-mediated constriction in HUV. Figure 3 C presents that the mRNA expression of BK_Ca_α was lower in frozen group, while the protein expression was higher compared with the control. No significant differences were found in mRNA and protein expressions of BK_Ca_β1 between both groups.

### Role of CACNA1C and CAMK2 in histamine-induced contraction

**Fig. 4:**
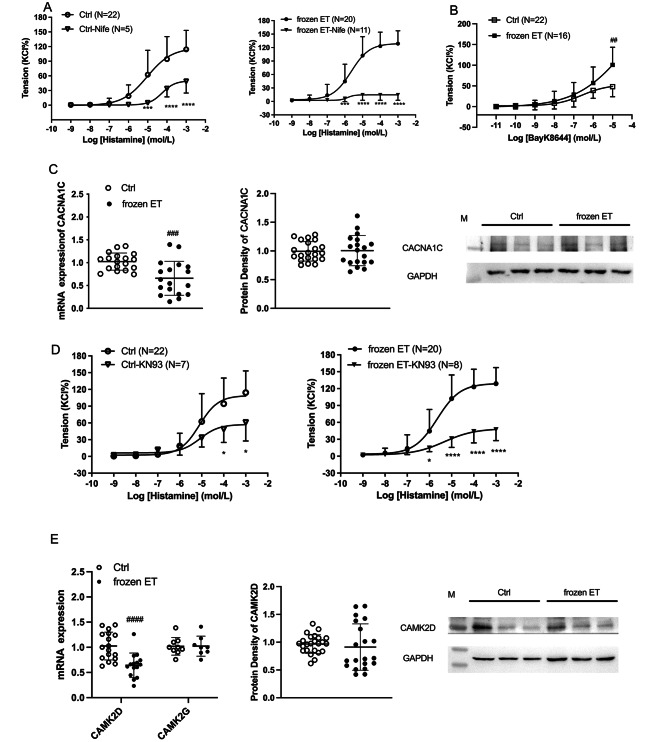
Functions of L-type voltage-dependent calcium channels and CAMK2 in histamine-induced vasoconstriction in HUV (**A**) The contractile responses to histamine with or without nifedipine (Nife, an L-type voltage-dependent calcium channel blocker, 10^− 5^ mol/L). (**B**) Bay K8644-mediated constriction in HUV. (**C**) The mRNA and protein expressions of CACNA1C. (**D**) The contractile responses to histamine with or without KN93 (a CAMK2 inhibitor, 10^− 5^ mol/L). (**E**) The mRNA expressions of CAMK2D and CAMK2G, and CAMK2D protein expression. Intra-group comparison, *, p < 0.05; ***, p < 0.001; ****, p < 0.0001. Inter-group comparison, ^##^, p < 0.01; ^###^, p < 0.001; ^####^, p < 0.0001

Figure 4A shows that histamine-induced vasoconstriction was significantly decreased by nifedipine in HUV, indicating thatCACNA1C were involved in histamine-induced contraction in HUV. The inhibitory of nifedipine was significantly stronger at the dose of histamine 10^− 6^ mol/L in frozen ET group in comparison to the control (Fig. 4A). The agonist for CACNA1C, Bay K8644-induced constriction in frozen ET group was stronger than that in the control (Fig. 4B). Figure 4 C presents that there was a decreased CACNA1C mRNA expression, although no significant change was found in its protein expression. The inhibition of KN93 was stronger at the dose of histamine 10^− 6^ mol/L and 10^− 5^ mol/L in frozen ET group than that in the control (Fig. 4D). The mRNA expression of CAMK2D was lower in the frozen ET than the control, and no remarkable difference was found in CAMK2G mRNA expression (Fig. 4E). The CAMK2D protein expressions were similar between the two groups (Fig. 4E). The unchanged CACNA1C and CAMK2D protein expression could not well explain the increased constriction caused by histamine or Bay K8644.

### Role of PKC and phenylephrine in HUV constriction

**Fig. 5:**
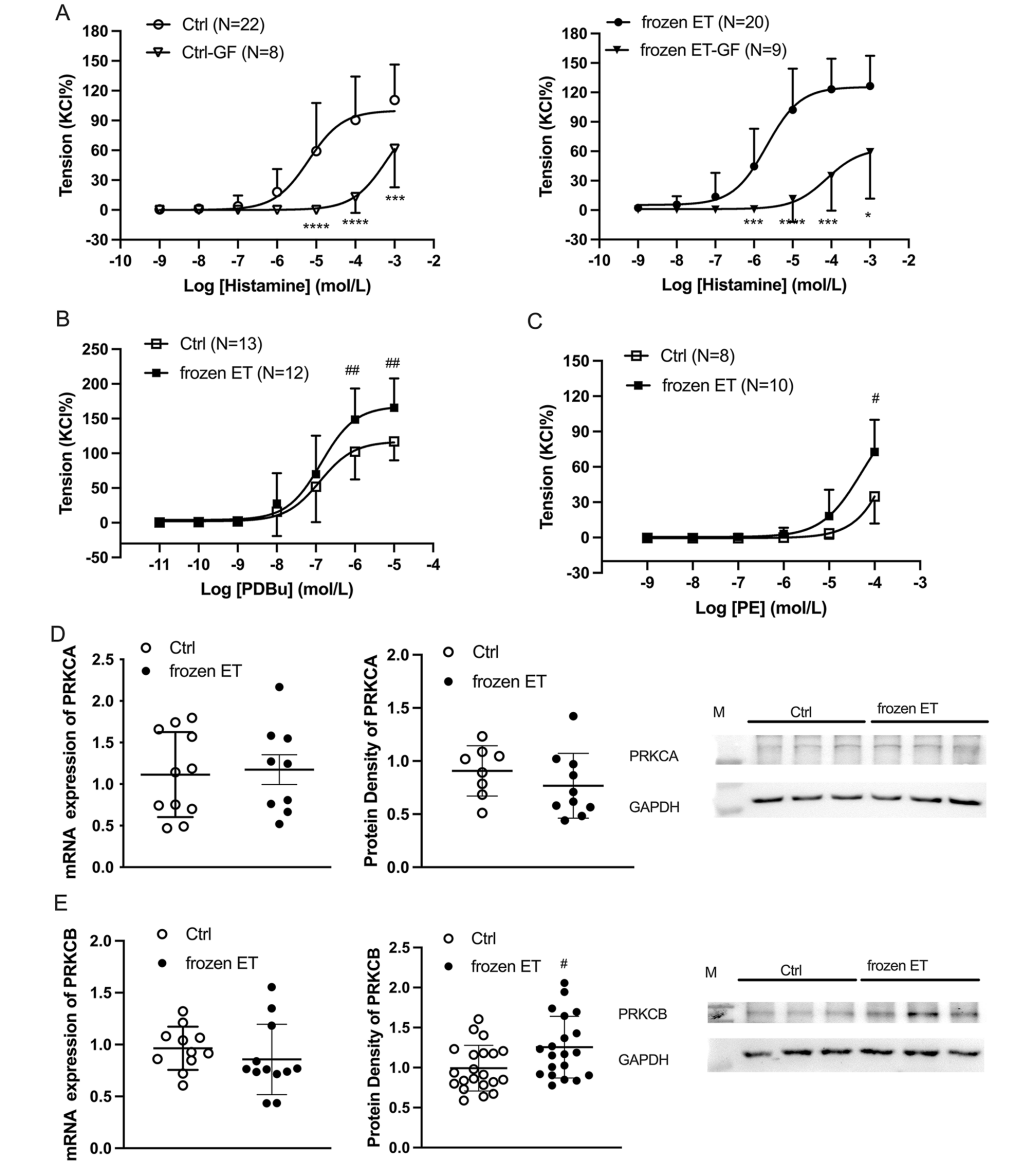
Role of phenylephrine and PKC in regulating vessel tone of HUV (**A**) The contractile responses to histamine with or without GF109203X (pan PKC inhibitor, GF, 10^− 6^ mol/L). (**B**) PDBu-mediated contraction in HUV. (**C**) Phenylephrine (PE)-induced constriction in HUV. (**D-E**) The mRNA and protein expressions of PKC (α and β). Intra-group comparison, *, p < 0.05; ***, p < 0.001; ****, p < 0.0001. Inter-group comparison, ^#^, p < 0.05; ^##^, p < 0.01

Pan PKC inhibitor GF109203X (GF) blocked the contractile responses to histamine in HUV from frozen ET and control group (Fig. 5A). In the presence of GF109203X, histamine-mediated constrictions were similar between both groups. The PKC agonist, PDBu potentiated vascular contraction in both groups and the constriction was stronger in frozen ET than that in the control (Fig. 5B). Phenylephrine (PE)-mediated vasoconstriction was greater in HUV from frozen ET than the control (Fig. 5C). Figure 5D and E show that no significant differences were found in PKCα mRNA and protein expressions as well as PKCβ mRNA expression. There was a remarkable increase in PKCβ protein expression in frozen ET when comparing with the control (Fig. 5E).

## Discussion

The present study proved that histamine-mediated contractile responses in human umbilical veins from pregnancy who received frozen embryo transfer was left-shift and the LogEC50 was lower in the frozen ET group when comparing with that in the control. In human umbilical veins, H1R played a predominant role in regulating vasoconstriction, while H2R showed little roles in either vascular constriction or dilation. BK_Ca_ and Kv did not obviously regulate histamine-mediated constriction in HUV. CACNA1C and CAMK2 participated in histamine-induced constriction of HUV, but their protein expressions were not increased in the frozen ET group. The functional responses of PKC and its protein expression of PKCβ were enhanced in the frozen ET group, which might be one of the reasons of increased contractile sensitivity to histamine in HUV.

In vitro fertilization-embryo transfer (IVF-ET) affects vessel tone of HUV. Recent studies reported that angiotensin II-mediated vasoconstrictions were greater in umbilical veins from pregnancy who received IVF-ET when comparing with the control [13]. But the contractile response to acetylcholine was lower in IVF-ET group [12]. However, previous vascular functional studies on HUV did not distinguish samples between fresh or frozen ET. The present study specially focused on possible influences of frozen ET on vessel tone of HUV and firstly demonstrated that human umbilical veins from the frozen ET pregnancy were more sensitive to histamine, especially at the doses of 10^− 6^ and 10^− 5^ mol/L). To the best of our knowledge, it was the first study to detect vascular effects of frozen ET on human umbilical veins.

Available evidences suggested that changes of histamine concentration in maternal plasma were involved in a number of complications from human pregnancy, for instance preeclampsia and preterm labor [14, 30, 31]. This study found that histamine concentration was increased in umbilical plasma from frozen ET when comparing with the normal pregnancy, which might influence vascular constriction or dilation in umbilical vascular systems. Notably, it is also the first work to compare the histamine concentrations in umbilical plasma between frozen ET and the normal pregnancies.

Histamine could cause sustained vasoconstriction in all HUV in the present study, consistent with the previous non-IVF related study [32]. The maximum contractile responses to histamine in HUV were also analogous to previous study [32]. Additionally, this study firstly revealed that histamine-mediated vasoconstriction in umbilical veins was mainly via H1R, in accordance with previous reports on umbilical arteries [18, 33] and other arteries [34]. H1R agonist induced similar vasoconstrictions to histamine (Supplementary figures), further demonstrating that H1R played a predominant role in regulating HUV constriction. Histamine did not dilate umbilical veins following the contraction by serotonin in both control and frozen ET group. The H2R antagonist did not significantly block histamine-induced constriction and its agonist did not obviously relax umbilical veins following application of serotonin, elucidating as the first time that H2R played very little roles on vessel tone of HUV. Interestingly, the important finding in the umbilical vein (the sole fetal blood vessel that can be obtained from healthy pregnancy for experiments), showed altered sensitivities in response to histamine, suggesting functional development of fetal vascular systems might be affected by frozen ET programming procedure. Whether the influence would be a benefit or negative impact in long-term is worth of further investigations in both clinical and basic science, in light of a number of studies having demonstrated that cardiovascular diseases could be initiated from prenatal insults during early developmental periods [35, 36].

In exploring possible mechanisms of frozen ET-induced changes, histamine receptors were tested. Molecular experiments showed decreased protein expressions of H1R and H2R in umbilical veins from frozen ET. The decreased H1R protein expression might be a negative feedback to the increased histamine concentration in umbilical plasma from frozen ET. The increased sensitivity to histamine might be due to the maybe enhanced downstream pathways after histamine binding with H1R in frozen ET.

It is well known that potassium channels are important regulators of vessel tone, and BK_Ca_ and K_V_ are the two main types of potassium channels in HUV [11]. However, the present study revealed that both BK_Ca_ and K_V_ played little roles in regulating histamine-mediated constriction in HUV from both groups. Besides potassium channels, calcium channels are also critical in control of vasoconstrictions [37]. This study found that histamine-induced vasoconstriction was significantly decreased by CACNA1C antagonist and CAMK2 inhibitor. The inhibition of nifedipine was remarkably greater in frozen ET than that of control in response to histamine at the dose of 10^− 6^ mol/L. Furthermore, CACNA1C agonist, BayK8644, potentiated the vasoconstriction in frozen ET group, indicating an increased activity of CACNA1C involved pathways. Furthermore, the inhibitory effects of KN93 was greater in frozen ET group than that of the control at the dose of histamine 10^− 6^ and 10^− 5^ mol/L, suggesting that the frozen ET might potentiate the pathways related to CAMK2 in HUV. However, frozen ET did not affect expressions of CACNA1C and CAMK2D. Therefore, the observed functional alterations in HUV due to frozen ET might be caused by changed activities of CACNA1C and CAMK2D or other mechanisms.

Calcium-sensitive pathways are commonly viewed as the reasons of increased vascular sensitivity, and those pathways include PKC route. Our subsequent experiments determined the PKC pathway and found there was an increase in PKC function and protein expression in frozen ET group. These new findings provide information in understanding the increased sensitivity to histamine in HUV from frozen ET. Besides functional testing on histamine, other vascular stimulator such as phenylephrine was determined in this study. The results demonstrated that phenylephrine-mediated contraction in frozen ET was also potentiated when comparing with control. Previous studies have shown that phenylephrine-mediated vasoconstriction also depended on PKC pathways [38].

Notably, the umbilical veins collected in this study were selected at fetal side, which are commonly viewed as fetal vessels. Since it is not ethically possible to test other vessels in normal human fetuses, HUV is good model in functional and molecular studies. In light of this, our findings regarding altered vessel tones in HUV indicates that fetal vessel development could be affected by frozen ET procedure, which definitely deserves further investigation.

## Conclusions

This study found as the first time that frozen ET procedure could cause an increase in contractile sensitivity to histamine in human umbilical veins, indicating that fetal vascular systems could be altered by frozen ET procedure in comparison to the normal pregnancy. A series of experiments revealed that the possible mechanism was increased PKC function and expression. Moreover, HUV is part of fetal vessels, and it can represent fetal vascular systems. Thus, the new data and findings in this study provide important insight into influences of frozen ET on development of fetal vessels and potential influences in long-term. However, it is still too early to conclude that the increased contractile sensitivity to histamine in umbilical veins is positive or negative to babies or life afterward. Considering numerous studies had demonstrated that cardiovascular diseases could be linked to abnormal development during embryo periods, the novel data gained may provide interesting clues to further investigating possible benefits as well as negative influence of frozen ET, which might improve health of babies from IVF-ET.

## Electronic supplementary material

Below is the link to the electronic supplementary material.

Supplementary Material 1

## Data Availability

The datasets used and/or analysed during the current study are available from the corresponding author upon reasonable request. Data regarding any of the subjects in the study has not been previously published unless specified. Data will be made available to the editors of the journal for review or query upon request.
