# Supplementary material for: In vitro fertilization with frozen embryo transfer increased histamine-mediated contractile sensitivity via PKCβ in human umbilical vein
Source: Reprod Biol Endocrinol. 2023 Jun 13;21:54. doi: 10.1186/s12958-023-01103-8 (PMC10262558; doi:10.1186/s12958-023-01103-8)
Supplement: Supplementary file 1 — Supplementary Material 1 [file 12958_2023_1103_MOESM1_ESM.doc]

Supplementary Material

**Supplementary Method**

The human umbilical veins (HUV) from control pregnancy were suspended in a 5 mL organ bath containing Krebs solution and measured with JZ101 isometric force transducer (Xinhangxingye Technology, Beijing, China) and recorded by Medlab6. Tissue baths were maintained at 37℃, and gassed continuously with a mixture of 95% O_2_ and 5% CO_2_. The rings were given 2 g of initial tension and allowed to equilibrate for 1 hour. Then the rings were stimulated with 120 mmol/L potassium chloride (KCl) to achieve maximal tension. Accumulative concentrations of histamine (HIS, 10^-9^-10^-3^ mol/L), 2-pyrdylethylamine dihydrochloride (2pyr, histamine 1 receptor agonist, 10^-9^-10^-3^ mol/L), were added into the organ bath to obtain the does-response curves.

**Supplementary Result**

Histamine and 2pyr induced similar doses-dependent constrictions in HUV.

## Supplementary Figures

120s

1g


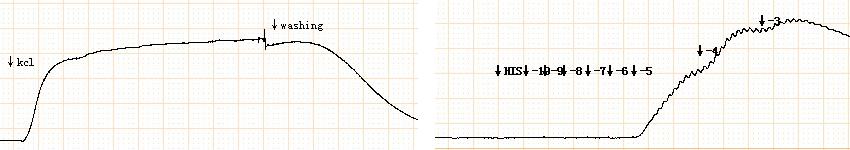
**Supplementary Figure 1.** Histamine (HIS) induced doses-dependent constrictions in normal HUV.

120s

1g


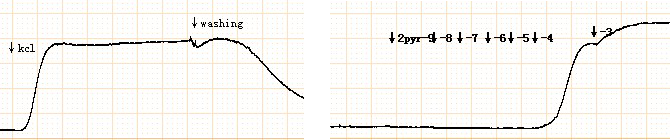


**Supplementary Figure 2.** Histamine 1 receptor agonist (2pyr) induced doses-dependent constrictions in normal HUV.
